# Prevent Frailty, Prevent Cardiovascular Disease: Early Identification of Patients at Risk

**DOI:** 10.1016/j.jacadv.2025.101701

**Published:** 2025-04-16

**Authors:** Naila Ijaz, Michael G. Nanna, Abdulla A. Damluji

**Affiliations:** a Thomas Jefferson University Hospital, Philadelphia, Pennsylvania, USA; b Yale School of Medicine, New Haven, Connecticut, USA; c Inova Center of Outcomes Research, Fairfax, Virginia, USA; d Johns Hopkins University School of Medicine, Baltimore, Maryland, USA

**Keywords:** cardiovascular disease, frailty, geriatric cardiology, HFpEF, prevention, sarcopenia

**Figure:**
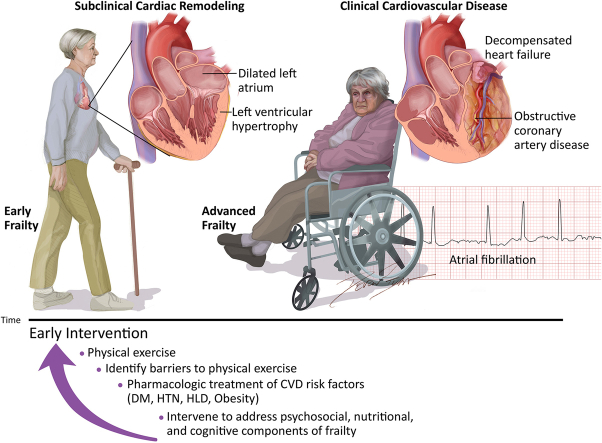

## The Bi-directional relationship between frailty and CVD

Frailty is a geriatric condition defined as an increased vulnerability to internal and external stressors. The most widely utilized instrument to diagnose physical frailty is the Fried physical frailty phenotype, when at least three of the five criteria are met: unintentional weight loss, weakness, exhaustion, slowness, and low physical activity.^1^ There are other types of frailty that lead to vulnerability, including cognitive frailty, nutritional frailty, and psychosocial frailty.^1^ Frailty prevention is highly relevant to the management of cardiovascular disease (CVD) because it increases the risk of incident CVD, hospitalization, institutionalization, and mortality. Additionally, it increases the risk of periprocedural complications in patients with CVD. CVD and frailty share risk factors and have similar molecular underpinnings, including systemic inflammation, metabolic dysfunction, and oxidative stress.^1^ They also share a bidirectional relationship, with the development of one causing the other. Frailty is reversible in its initial stages, hence it is important to identify and intervene. Interventions to reverse frailty are an area of ongoing research.

## Frailty awareness and detection

**Figure 1:**
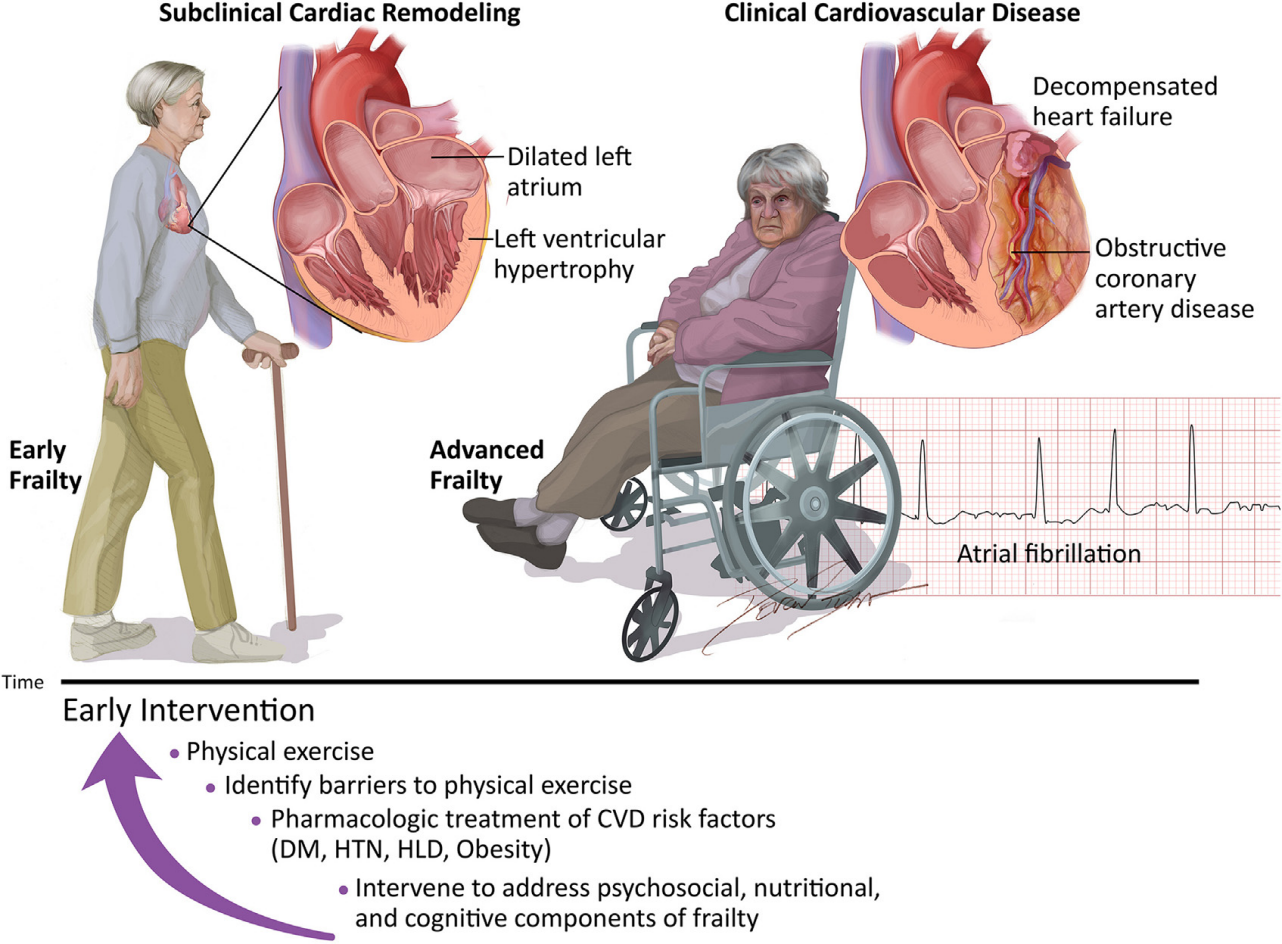
**Early Interventions Can Prevent Progression of Frailty and Cardiovascular Disease** Frailty is associated with subclinical cardiovascular remodeling including left atrial dilation, greater left ventricular mass index, worse diastolic function, and higher E/e' ratio. Without appropriate interventions, these patients develop progressive frailty and cardiovascular disease, including obstructive coronary artery disease, atrial fibrillation, and decompensated heart failure. Early interventions that include physical exercise, identifying barriers to exercise, pharmacologic treatment of risk factors, that is, diabetes, hypertension, hyperlipidemia, and obesity, and intervening with psychosocial, nutritional, and cognitive interventions, may reverse or halt the progression of frailty and prevent cardiovascular disease. CVD = cardiovascular disease; DM = diabetes mellitus; HLD = hyperlipidemia; HTN = hypertension.

In cardiovascular practice, patients frequently present after they have developed CVD and are at risk for developing frailty or have already developed both. Hence, knowledge of how to prevent frailty and counseling patients on this subject is key to helping them maintain good cardiovascular health. Exercise, lipid management, blood pressure control, and diabetes management have been associated with prevention of physical frailty, just as they are associated with prevention of CVD.^2^ The association between frailty and CVD has not only been proven by studies on cardiovascular outcomes, but there is also evidence that frailty is associated with cardiac structural changes that can be visualized on echocardiography that predate clinical CVD and have been associated with the development and progression of frailty. A recent study from the Atherosclerosis Risk in Communities cohort demonstrated that the presence of a greater left ventricular mass index, worse diastolic function, and higher E/e' ratio was associated with a greater likelihood of progressing to a worse frailty status over a 5-year follow-up and similarly, frailty was associated with similar echocardiographic changes.^3^ This study showed a bidirectional association between frailty and left ventricular remodeling that predates heart failure with preserved ejection fraction (HFpEF) among older adults and the researchers proposed that interventions to modify one may exert beneficial effects on the other. Studies have also shown that patients with HFpEF are frequently affected by other comorbidities: chronic obstructive pulmonary disease, diabetes, anemia, and obesity.^4^ In many patients, these may be driving their frailty status. Hence, in frail and prefrail patients, identifying the contributing factors, and intervening to treat physical frailty is essential to preventing CVD (Figure 1). Interventions to address frailty may prevent or delay the development of clinical HFpEF and coronary artery disease.

## Frailty interventions and barriers to care

Of the many nutritional, pharmacological, and exercise-based interventions studied to reverse frailty, it was found that exercise-based multimodal rehabilitation interventions were the most promising in reversing frailty or preventing its progression.^1^ Exercise improves inflammation, decreases the risk of oxidative damage, improves mitochondrial function, and improves insulin sensitivity.^1^ In these ways, it may prevent vascular changes that result in intima-media thickening and increased arterial stiffness, which has been found to be associated with sarcopenia. Exercise therapy has been proven to reverse markers of frailty in patients with CVD, even those recently admitted with HFpEF exacerbations. REHAB-HF (Rehabilitation Therapy in Older Acute Heart Failure Patients) showed improvement in markers of physical frailty (short physical performance battery, 6-minute walk distance, and frailty status as measured by modified Fried criteria) in patients with acute decompensated heart failure who were enrolled in an early, tailored, progressive physical rehabilitation program developed for frail older adults with acute decompensated heart failure.^5^ However, older adults are frequently affected by conditions that prevent participation in exercise. In a systematic review of 25 qualitative studies assessing factors that influence physical activity in older adults, few factors that were found were slowness and fatigue, alluding to the vicious cycle that frailty can put individuals into, causing progressive frailty.^6^ Additional factors included arthritis, obesity, dizziness, drowsiness, environment, perception of capabilities, and fear of falls. Dizziness and drowsiness may be due to polypharmacy, which is frequently overlooked in these patients. Not all adults may have access to a safe physical environment for exercise; hence, case managers should be engaged to identify community resources. In those frail and prefrail patients with CVD, cardiac rehabilitation should be offered and not only include a supervised exercise intervention but also help to identify and address barriers to exercise in this population.

There is increasing recognition of the various subtypes of frailty other than physical frailty, which include nutritional frailty, psychosocial frailty, and cognitive frailty. Studies have shown that addition of social support interventions and nutritional interventions (whey protein supplementation) and cognitive training exercises have resulted in frailty reduction.^1^ Whereas not every intervention may be required for every patient, that is, patients with chronic kidney disease should not be on a high-protein diet, patients should be screened for risk factors that put them at risk for these subtypes of frailty so that appropriate interventions can be identified. Cognitive frailty also has similar underpinnings to physical frailty and CVD, hence addressing vascular risk factors can prevent or halt cognitive decline.^7^

## HFpEF and obesity: an underrecognized subgroup at high frailty risk

A frequently underrecognized cohort at high risk for developing frailty and subsequent poor outcomes are patients with HFpEF and obesity. Frailty and HFpEF are closely related with 60% to 90% of patients with chronic stable HFpEF identifying as frail.^8^ Obesity is a major risk factor for younger-onset HFpEF. These patients are not typically identified as being at high risk for frailty or being frail because they are not underweight but are at high risk for developing limited functional status and other sequelae from frailty. Hence, interventions to modify risk factors and prevent or reverse HFpEF are essential for the prevention of frailty. There have been small advances in pharmacotherapy to improve functional status in HFpEF patients, with the PRESERVED-HF (Effects of Dapagliflozin on Biomarkers, Symptoms and Functional Status in Patients with Preserved Ejection Fraction Heart Failure) study showing use of dapagliflozain, a sodium-glucose transporter 2 inhibitor, is associated with improvement in 6-minute walk distance.^9^ More recently, a glucagon-like peptide-1 receptor agonist has proven to improve cardiovascular outcomes in patients with obesity and HFpEF. The SUMMIT (Tirzepatide for Heart Failure with Preserved Ejection Fraction and Obesity) trial studied tirzepatide treatment in patients with HFpEF and obesity with a BMI ≥30 kg/m^2^.^10^ Tirzepatide is a glucagon-like peptide-1 agonist that has been proven to be effective for weight loss. Results not only showed that patients treated with tirzepatide had a lower risk of primary composite outcome (major adverse heart failure outcomes, death from cardiovascular causes, and worsening heart failure events) but also demonstrated an improvement in health status, exercise tolerance, and a decrease in high-sensitivity C-reactive protein level, a marker of systemic inflammation. It is important to note that the participants in this trial had an average age of 65 years and the oldest participants were 75 years of age. Additionally, the reduction in inflammation as evident by biomarker analysis suggests improvement in the physiological processes that cause HFpEF, coronary artery disease, and frailty, making this an important intervention for healthy cardiovascular aging and maintenance of functional status.

## Functional independence as a preventative health goal

Whereas it is important to counsel patients on healthy behaviors to optimize their cardiovascular health, that is exercise, lipid management, blood pressure management, diabetes management, it may be more motivating to adopt these behaviors when patients are counseled that optimizing their vascular health with these interventions will decrease their risk of developing physical frailty. Whereas it may require some scientific knowledge to understand the implications of CVD, it may be easier to comprehend and more motivating to understand the concepts of maintaining functional independence and preventing institutionalization. Hence, when older adults are counseled on CVD prevention, frailty prevention should also be a part of that discussion.

## Conclusions

As the average life expectancy is increasing, the prevalence of geriatric syndromes is increasing, which make aging not only burdensome for older adults but also their family members and adds significant financial implications on the health care system. Physical frailty leads to reduced patient independence, higher institutionalization rate, and exacerbate chronic conditions including CVD, resulting in frequent hospitalizations. Therefore, early detection of frailty followed by interventions to prevent or reverse frailty may positively influence the incidence and progression of CVD in older patients. Frailty that predates CVD may be caused by various factors including obesity, arthritis that limits exercise participation, drowsiness due to polypharmacy, and unsafe environment for physical activity. These factors need to be identified and addressed early to help patients age gracefully by preventing frailty and CVD.

**KEY POINTS**

• Frailty and CVD share a bidirectional relationship with similar underpinnings: inflammation and metabolic dysregulation.
• Optimizing risk factors for CVD, that is, obesity management, diabetes and blood pressure control, management of hyperlipidemia, and identifying barriers to therapy, including exercise, can prevent or reverse frailty in addition to delaying or preventing the onset of clinical CVD.
• It is important to recognize frailty subtypes apart from physical frailty, that is, nutritional, psychosocial, and cognitive frailty, so that appropriate interventions can be recommended.
• Older adults may find it more motivating to adhere to recommendations for CVD prevention when also put in context of preventing physical frailty and functional dependence.

## Funding support and author disclosures

This work was supported by mentored patientoriented research career development award from the 10.13039/100000050National Heart, Lung, and Blood Institute K23-HL153771. Dr Nanna has received unrelated current research support from the 10.13039/100005485American College of Cardiology Foundation supported by the George F. and Ann Harris Bellows Foundation, the 10.13039/100006093Patient-Centered Outcomes Research Institute (PCORI), the Yale Claude D. Pepper Older Americans Independence Center (P30AG021342), and the 10.13039/100000049National Institute on Aging (K76AG088428); and has received personal fees from Heartflow, Inc, Merck, and Novo Nordisk. Dr Damluji has received research funding from the Pepper Scholars Program of the Johns Hopkins University Claude D. Pepper Older Americans Independence Center funded by the 10.13039/100000049National Institute on Aging P30-AG021334; mentored patient-oriented research career development award from the 10.13039/100000050National Heart, Lung, and Blood Institute K23-HL153771; the NIH 10.13039/100000049National Institute of Aging R01-AG078153, and the 10.13039/100006093Patient-Centered Outcomes Research Institute (PCORI). Dr Ijaz has reported that she has no relationships relevant to the contents of this paper to disclose.
